# Video-Based Stress Detection through Deep Learning

**DOI:** 10.3390/s20195552

**Published:** 2020-09-28

**Authors:** Huijun Zhang, Ling Feng, Ningyun Li, Zhanyu Jin, Lei Cao

**Affiliations:** Department of Computer Science and Technology, Centre for Computational Mental Healthcare, Research Institute of Data Science, Tsinghua University, Beijing 100084, China; fengling@tsinghua.edu.cn (L.F.); liny18@mails.tsinghua.edu.cn (N.L.); jinzy15@tsinghua.org.cn (Z.J.); cao-l17@mails.tsinghua.edu.cn (L.C.)

**Keywords:** stress detection, video, facial expression, action, deep learning

## Abstract

Stress has become an increasingly serious problem in the current society, threatening mankind’s well-beings. With the ubiquitous deployment of video cameras in surroundings, detecting stress based on the contact-free camera sensors becomes a cost-effective and mass-reaching way without interference of artificial traits and factors. In this study, we leverage users’ facial expressions and action motions in the video and present a two-leveled stress detection network (TSDNet). TSDNet firstly learns face- and action-level representations separately, and then fuses the results through a stream weighted integrator with local and global attention for stress identification. To evaluate the performance of TSDNet, we constructed a video dataset containing 2092 labeled video clips, and the experimental results on the built dataset show that: (1) TSDNet outperformed the hand-crafted feature engineering approaches with detection accuracy 85.42% and F1-Score 85.28%, demonstrating the feasibility and effectiveness of using deep learning to analyze one’s face and action motions; and (2) considering both facial expressions and action motions could improve detection accuracy and F1-Score of that considering only face or action method by over 7%.

## 1. Introduction

Stress has become more and more widespread and severe in the modern society. Stress that is left unchecked and handled could contribute to many health problems, threatening people’s feelings, thoughts, behaviors, and well-being. Being able to detect stress can help people take active steps to manage the stress before bad consequences are incurred.

Traditional stress detection relies on psychological questionnaires [1] or professional psychological consultation [2]. As the results of questionnaires depend largely on the answers given by individuals, the stress measure is quite subjective. When people choose to express their psychological states with reservations, the result scale would be biased. To overcome the limitations of the questionnaire surveys, the methods of automatically detecting stress by sensing an individual’s physical activities through wearable devices such as mobile phones with embedded sensors [3,4,5,6,7,8] or based on physiological signals such as heart rate variability HRV, electrocardiogram ECG, galvanic skin response GSR, blood pressure, electromyogram, electroencephalogram EEG, etc. from dedicated sensors [9,10,11,12] have been developed. While these methods are able to objectively sense people’s stress states, they usually demand wearable equipments and sensors, which could hardly realize contact-free measurement.

Currently, the ubiquitous deployment of contact-free video cameras in surroundings, together with the rapid progress of data collection and analysis techniques, offers us another channel to detect one’s stress based on image sequences captured from a monitoring video camera. Compared with previous sensory devices, the later offers the following three benefits. First, it is more convenient, particularly in places like schools, hospitals, and restricted areas like prisons, where no carry-on devices are needed or allowed. Second, it has a very long standby time and can easily reach mass audience at a very low-cost. Third, the continuous frames it captures enable us to grasp and analyze people’s stressful states more naturally without interference of artificial traits and factors.

The aim of this study is to leverage contact-free video cameras for stress detection.

There are several recent studies reporting findings that facial signs and expressions can provide insights into the identification of stress [13,14,15], and symptoms of stress are usually linked with fluctuations in physiological signals (e.g., heart rate, blood pressure, galvanic skin response, etc.) and physical activities [16]. Most of the existing work in the literature focused on extracting facial signs such as mouth activity, head motion, heart rate, blink rate, gaze spatial distribution, pupil dilation, and eye movements from different facial regions [13,17,18], or used the Facial Action Coding System (FACS) [19] and extracted Action Units (AUs) from the face frames for stress detection [20,21].

**Figure 1:**
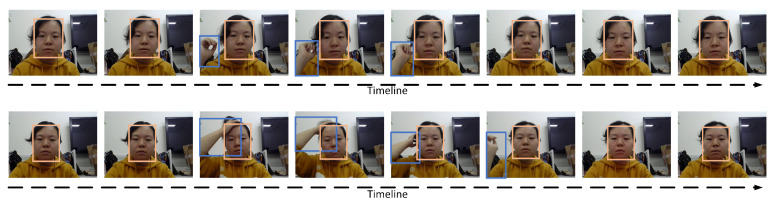
Two image sequences of the same person when watching an unstressed video clip (**upper**) and stressed video clip (**lower**).

Deep learning has been widely and successfully applied in many fields such as computer vision, emotion analysis and so on. Different from the existing work which extracted the features through hand-crafted feature engineering methods, in this work, we conduct stress detection through deep learning of features’ representations. Furthermore, beyond facial regions analysis, we leverage and integrate user’s action cues to enhance the video-based stress detection. The rationale could be glimpsed from Figure 1, which shows two image sequences of the same person watching an unstressed video clip (upper) and stressed video clip (lower), respectively. The facial expressions of the person under two states are very similar, but her action motions offer some clues for the discrimination of the stressful state. The subject touched the ear unconsciously when unstressed, but grabbed the hair above the head when she felt stressed. In this case, action motions and facial expressions could complement each other with valid information contributing to stress detection.

To this end, we proposed a Two-leveled Stress Detection Network (TSDNet), which firstly learns face- and action-level representations separately, and then fuses the results through a stream weighted integrator for stress identification.

To address the challenge that images manifesting subject’s stressed states usually hide in a long sequence of image frames with subtle distinctions, in addition to fusing actions and facial expressions, we designed a number of attention mechanisms, including face-level multi-scaled pooling attention, action-level frame attention, aiming to capture affective facial expressions and action motions from the video. A stream weighted integrator with local and global attention was also implanted to strengthen the detection performance.

Overall, the contributions of the paper can be summarized as follows.

- We presented a two-leveled stress detection network (TSDNet), which learns to fuse facial expressions and action motions in videos for stress detection.
- A set of attention mechanisms were particularly designed to capture affective facial expressions and action motions from the video, and integrate the results with local and global attention.
- A video dataset containing 2092 labeled video clips was constructed. The experimental results on the built dataset showed that: (1) TSDNet outperformed the hand-crafted feature engineering approaches with detection accuracy 85.42% and F1-Score 85.28%, demonstrating the feasibility and effectiveness of using deep learning to analyze one’s face and action motions; (2) considering both facial expressions and action motions could effectively improve detection accuracy and F1-Score of that considering only face or action method by over 7%.

The remainder of the paper is organized as follows. In Section 2, we provide relevant related work on stress detection. In Section 3, we describe materials and our method of video-based stress detection. We evaluate the performance of the proposed method in Section 4, conclude the paper with a brief discussion of future work in Section 5.

## 2. Related Work

In this section, we review some closely related work on image-based and video-based stress detection.

### 2.1. Image-Based Stress Detection

Observing that the signs of stress could be more easily detected by looking at the condition of the face, particularly the lines or wrinkles around the nose, mouth, and eyes, [22,23] investigated three facial parts (the eyes, nose and mouth) which are significant for stress detection. [23] extracted Gabor filter and HOG (Histogram of Oriented Gradients) features from each part of the face in pixels through visual image encoding process, and fed them into three different SVM classifiers. The obtained three results were then fed into slant binary tree to get the final results. Experiments were performed on the ten-women JAFFE dataset, where each subject has a stress expression image and a neutral expression image [24]. The experimental result shows that the nose is a part of the face that mostly indicates stress, and about 86.7% of detection accuracy can be achieved. Along the same line [22] extracted relevant facial features from an image pixel using DoG (Difference of Gaussians), HOG, and DWT (Discrete Wavelet Transform) histogram methods, and then combined and reconstructed the obtained multi-histogram features into global features. A Convolutional Neural Network with three convolutional layers and two max-pooling layers was trained on the color FERET face database. The stress recognition accuracy reached about 95%.

As symptoms of stress are usually linked with fluctuations in physiological (e.g., heart rate, blood pressure, galvanic skin response, etc.) and physical activities [16], such facial features like gaze spatial distribution, saccadic eye movement, pupil dilation, and blink rate, etc., were utilized to distinguish stress levels. In [25], the authors detected stress and anxiety based on a set of facial signs, including mouth activity, head motion, heart rate, blink rate, and eye movements. Methods used for extracting these features from different facial regions were discussed and the performance was tested on a data set containing 23 subjects.

### 2.2. Video-Based Stress Detection

#### 2.2.1. Facial Cues Based

Ref. [13] extended the previous image-based stress detection work, and proposed a stress and anxiety analysis framework based on facial cues recorded from videos. It extracted four groups of features (eyes related features, mouth related features, head movements and heart rate) from facial videos, and further analyzed the correlation between facial parameters and the amount of stress/anxiety perceived by the participants. The experiment results showed that the four groups of facial cues including eyes related features, mouth activity, head movements and heart rate were effective for stress/anxiety classification and could well discriminate stress and anxiety.

Based on the findings that mouth activities correlate with signs of psychophysical status, [17] developed a semi-automated algorithm to extract mouth activity from videos. The algorithm utilized Eigen-features and template-matching to classify mouth actions. The performance of the proposed mouth action classification algorithm was evaluated on a dataset containing 25 subjects, the classification accuracy could reach 89%. Furthermore, the proposed algorithm was evaluated for stress/anxiety assessment. The tests on 23 participants demonstrated that the stressed/anxious participants were more likely to open mouth and their openness intensity was greater.

Ref. [18] developed a real-time non-intrusive monitoring system, which detected two stress related emotional states (anger and disgust) of the driver from facial expressions. It used a near-infrared camera on the dashboard to capture the near frontal view of the driver’s face. The developed system consisted of two parts. The first part was face acquisition module, which detected and tracked the drivers’ faces and captured the facial landmarks. The second part was stress detection module, where a pre-trained emotion detection model was applied to detect the facial expressions and then the frame level expressions were integrated to determine the stress of the driver on sequence level. The experiments on the two recorded datasets (one was recorded in an office and the other is recorded in a car) showed that the system can reach 90.5% accuracy for in-door tests and 85% accuracy for in-car tests [18].

#### 2.2.2. Facial Action Units (AUs) Based

Ref. [20,21] used the Facial Action Coding System (FACS) to extract Action Units (AUs) from the face frame for stress detection. As known, FACS [19] divides the face into 46 primary action units (AUs) from upper-level to lower-level. Under the assumption that each emotion is associated with different facial muscle patterns, FACS determines the emotions of the individual by analyzing facial regions where these muscles are activated.

Ref. [20] examined five one-hour long videos. Each video was about a subject who was typing, resting, and exposed to a stressor task (i.e., a multitasking exercise combined with social evaluation). Then, 17 different Action Units (AUs) like Inner Brow Raiser, Brow Lowerer and Dimpler were extracted from upper-level to lower-level face frame-wise. Based on the extracted features, four classical machine learning methods (i.e., Random Forest, LDA, Gaussian Naive Bayes and Decision Tree) were employed to detect mental stress. The experimental result showed that the proposed AUs-based approach was able to achieve an accuracy of up to 74% in subject independent classification and 91% in subject dependent classification, indicating that the AUs which are most relevant for stress detection are not consistently the same for all 5 subjects, and using facial cues, a strong person-specific component was found during classification.

Ref. [21] decided Depression Anxiety Stress Scale (DASS) levels based on 31 AUs extracted through FACS and a three-layered noninvasive architecture. The first layer normalized the video frames, and classified the extracted AUs using a method based on Active Appearance Models (AAM) and a set of multi-class Support Vector Machines (SVMs). The second layer built a matrix based on the intensity levels of the selected AUs. Finally, obtaining the matrix output from the second layer, the third layer employed a neural network to analyze the patterns and predict the DASS levels (Normal, Mild, Moderate, Severe, or Extremely Severe) for each of the three emotional states (depression, anxiety, and stress). The experimental results showed that the method can achieve 87.2% accuracy for depression, 77.9% for anxiety, and 90.2% for stress.

#### 2.2.3. Fusion of Visual and Thermal Spectrums for Stress Recognition

Inspired by the research results that stress could be successfully detected from thermal imaging due to changes in skin temperature under stress [26], as well as the successful use of both thermal spectrum (TS) and visible spectrum (VS) imaging in modeling, analyzing, and recognizing facial expressions [27,28,29,30,31,32,33,34] proposed a stress recognition method by fusing visual and thermal spectrums of spatio-temporal facial data. A temporal TS and VS video database ANUStressDB, containing videos of 35 subjects watching stressful and non-stressful film clips, was proposed for stress recognition. It used a hybrid of a genetic algorithm (GA) and SVM to select salient divisions of facial block regions and decide whether using the block regions can enhance the performance of stress recognition. The experimental results showed that compared with the stress recognition performance using VS or TS videos independently, there is an obvious improvement after using the fusion of facial patterns from VS and TS videos. Moreover, the genetic algorithm selection method led to better performance than using all the facial block divisions. The best performance was obtained from HDTP (dynamic thermal patterns in histograms) features fused with LBP-TOP (local binary patterns on three orthogonal planes) features for TS and VS videos using a hybrid of a genetic algorithm and a SVM, achieving a 86% accuracy.

Furthermore, [35] further extended the work by representing a thermal image as a group of super-pixels, and extracting the features from thermal super-pixels rather than from pixels directly as done in [34]. According to [36], Super-pixel (a group of adjacent pixels which have similar characteristics and special information) representation has been used for face recognition. Besides, a thermal super-pixel is thus a group of pixels with similar color (temperature) which seems like a more natural representation for thermal images as compared to dividing images into non-overlapping blocks. In this way, with highly correlated adjacent pixels grouped together, the effectiveness of stress recognition can be improved and the processing speed has also been increased. The experimental results on ANUstressDB database showed that the method outperformed [34], achieving a 89% classification accuracy.

The work reported here differs from the previous work in the following two aspects. Firstly, we took a deep learning strategy to avoid the labor-intensive hand-crafted feature engineering approach. Secondly, besides facial regions analysis, we employed user’s action cues to enhance the detection performance. A stream weighted integration method embedded with local and global attention mechanisms was particularly designed and evaluated.

## 3. Materials and Methods

### 3.1. Data Collection

We invited 122 volunteers (58 males and 64 females of age 18–26) to participant in our study. The participants are college students from eight universities located in three different places (Beijing, Harbin, and Shanghai) in China. A Participant Consent Form was issued and signed by each participant before the study.

Preparation for Data Collection. There are many kinds of stressors that may stimulate stress. Playing computer games [37,38], answering difficult questions [39], and solving difficult problems [40] are some example stressors. In this work, we referred to the method of using infrared cameras to record the affective reactions (neutral, relaxed, and stressed) of the participants when they watched three different types of 2-min video clips [25,40]. The neutral video clips were about scenery or food making. The relaxed ones were highlights of variety show. The stressed ones were science programs with rich knowledge. Each scientific program was followed by a question-answering test. Each test contained ten questions. Half of them were multiple choices and the other half were blank fillings. The total score was 100. We designed the questions in such a way that it was very hard to come up with the correct answers unless the participants could understand the content and grasp the knowledge points well enough in the video. To stimulate the cognitive stress a bit, before the test, we announced to the participants that they could get some extra rewards if achieving test scores over 50 as incentive.

Procedure of Data Collection. We let the participants firstly watch a relaxed video clip followed by a neutral one with 10 s as a break in between. Before playing the third science video clip, we guided the participants to go through the follow-up test questions for 30 s in advance, and completed the online tests after watching the video clip.

We developed an application tool to automatically collect and save the videos of the participants when they watched the three types of video clips. Correspondingly, each obtained video lasted for 2 min. The videos collected while the participants watching the relaxed and neutral video clips were labeled “unstressed”, and “stressed” otherwise.

Pre-Processing of Collected Video Data. We collected totally 490 videos about the participants. The total duration of the collected videos was 2 h 38 min 52 s. The frame rate of the camera used is 30 fps.

We dropped the collected videos which failed to capture the faces due to the misaligned camera or the dim ambient light, or had the short recording time due to the abnormal program exit.

To cut down the training time, we partitioned each video into eight 15-s samples. If the last sample was less than 15 s, we appended it with its precedent sample. In this way, we acquired 2092 video samples, including 920 labeled “stressed” and 1172 labeled “unstressed”.

We randomly split the subjects into three groups, where 60% of the subjects for training, 20% of the subjects for validation, and the rest 20% of the subjects for testing. Especially, to obtain the more reliable results, we did three divisions and calculated the average results. The numbers of segmented 15-s video samples used for training, validation, and testing are given in Table 1.

We further resized all the input images (including face images, still images, and optical flows) to 70 × 70 pixels. To prevent over-fitting, we conducted a random 64 × 64 cropping and normalization to the training images, and a center-around 64 × 64 cropping and normalization to the validation and testing images.

### 3.2. Framework

The task of our video based stress detection is to sense the affective state (stressed or unstressed) of a user based on his/her video data $V=(frame_{1},frame_{2},\cdots ,frame_{n})$, where $frame_{1},frame_{2},\cdots ,frame_{n}$ is a sequence of image frames of the video.

The proposed model TSDNet firstly learns face- and action-level representations separately, and then fuses the results through a stream weighted integrator with local and global attention for stress identification.

#### 3.2.1. Face-Level Representation Learning

The learning of the face-level representation proceeds in three steps.

Step 1: Localize the face region in each frame of the video.

We adopted the technique [41] to automatically extract the face region in each frame, and then invited 5 volunteers to manually check the obtained face images. Let FaceSeq(V) = $\left\{face_{1},face_{2},\cdots ,face_{n}\right\}$ denote a sequence of face images framed from *V*.

Step 2: Identify the key face images from the sequence of face images.

Considering the subtle differences among the face images in the video, to capture affective expressions hidden in the sequence of similar face images and strengthen the detection performance, we identified two key face images (the most expressive face image and the most expressionless face image) from the sequence of face images. Their distance would be served as the basis for stress detection in the next Step 3.

We turned the identification of these two key face images into a binary classification and sorting problem. For each face image, we expected to obtain the probability eProb(·) that represents whether this face is expressive or not.

We built an expression classifier to discriminate expressive and expressionless face images based on Resnet [42]. We trained the expression classification network on the modified FER2013 dataset [43]. FER2013 is the dataset for facial expression recognition. It contains 7 labels (i.e., “angry”, “disgust”, “fear”, “happy”, “sad”, “surprise”, “neutral”). We kept the data labeled “neutral" as “expressionless” and regarded the other six kinds of labels as “expressive”.

We fed each face image $face_{1},$$face_{2},$$\cdots ,face_{n}\in FaceSeq\left(V\right)$ into the pre-trained binary expression classification model, and got the probability $eProb(face_{1}),$$eProb(face_{2}),$$\cdots ,eProb(face_{n})$. We sorted the probabilities in an descending order, and selected the corresponding first and last face image as the most expressive face image (denoted as $face_{e}$) and most expressionless face image (denoted as $face_{l}$).

Step 3: Learn the face-level representation.

The face level learning of one’s affective state was based on the difference between the most expressive and the most expressionless face images. Apart from the multi-scaled fine and coarse grained differences exploration, we also investigated possible difference correlations between the two images. Through the thorough and extensive comparison of the most expressive and expressionless face images, we established the face level representation for stressful state detection.

(1) Computing the Fine-Grained Difference

Applying two parameter-shared Resnets to face image $face_{e}$ and $face_{l}$, we acquired their basic feature maps $Resnet(face_{e})$ and $Resnet(face_{l})$ in the domain of $R^{C\times H\times W}$, where *C*, *H*, and *W* represent the channel number, height, and weight, respectively. In the study, C=512, H=8, and W=8.

We computed their fine-grained difference $D_{0}\left(face_{e},face_{l}\right)$ via an element-wise minus operation:

(1) $$D_{0}\left(face_{e},face_{l}\right)=Resnet\left(face_{e}\right)-Resnet\left(face_{l}\right)\;\;\in \;R^{C\times H\times W}$$

To learn the difference further, we fed $D_{0}\left(face_{e},face_{l}\right)$ into a residual block, consisting of a two-convolution layer, a Batch Normalization layer, and an active function (i.e., ReLU function), and obtained output $D(face_{e},face_{l})$ with residual connection.

(2) Computing the Coarse-Grained Differences

To target at high-level differences covering multiple regions of the face, we rolled up from the basic fine-grained difference between $face_{e}$ and $face_{l}$, and derived coarse-grained differences through a multi-scale pooling operation with a two-layered attention mechanism.

**Figure 2:**
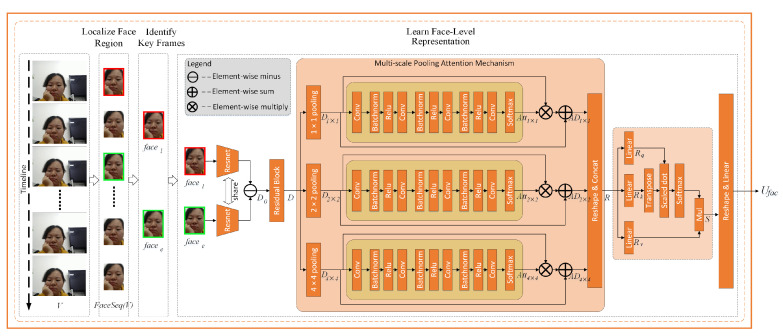
Face-level representation learning.

As shown in Figure 2, an average pooling with kernel size of (1 × 1), (2 × 2), and (4 × 4) was enforced on $D(face_{e},face_{l})$, generating three coarse-grained differences $D_{1\times 1}\in R^{C\times H\times W},\;D_{2\times 2}\in R^{C\times \frac{H}{2}\times \frac{W}{2}}$, and $D_{4\times 4}\in R^{C\times \frac{H}{4}\times \frac{W}{4}}$, respectively.

To learning the influential distribution of each coarse-grained metric, an attention block using convolutional layers, batch normalization layers, and ReLU function layers with Softmax function was designed, and obtained the attention distribution feature maps $Att_{1\times 1}\in R^{C\times H\times W}$, $Att_{2\times 2}\in R^{C\times \frac{H}{2}\times \frac{W}{2}}$, and $Att_{4\times 4}\in R^{C\times \frac{H}{4}\times \frac{W}{4}}$.

(2) $$\begin{array}{c} AttB(\cdot )=Conv(ReLU(BatchNorm(\cdot ))) \end{array}$$

(3) $$\begin{array}{c} Att_{1\times 1}=Softmax\left(AttB\left(AttB\left(AttB\left(Conv\left(D_{1\times 1}\right)\right)\right)\right)\right)\\ Att_{2\times 2}=Softmax\left(AttB\left(AttB\left(AttB\left(Conv\left(D_{2\times 2}\right)\right)\right)\right)\right)\\ Att_{4\times 4}=Softmax\left(AttB\left(AttB\left(AttB\left(Conv\left(D_{4\times 4}\right)\right)\right)\right)\right) \end{array}$$

We employed element-wise multiply to the attention distribution feature maps and the original post-pooling feature maps, mapping the attention distribution back to the post-pooling feature maps.

(4) $$\begin{array}{c} AD_{1\times 1}=D_{1\times 1}\times Att_{1\times 1}+D_{1\times 1}\\ AD_{2\times 2}=D_{2\times 2}\times Att_{2\times 2}+D_{2\times 2}\\ AD_{4\times 4}=D_{4\times 4}\times Att_{4\times 4}+D_{4\times 4} \end{array}$$

For ease of computation, we reshaped $AD_{1\times 1}$, $AD_{2\times 2}$, and $AD_{4\times 4}$ into two dimensions, i.e.,

$$AD_{1\times 1}\in R^{C\times H\times W}\overset{\mathrm{reshape}}{⟶}AD_{1\times 1}^{\prime }\in R^{C\times HW},$$

$$AD_{2\times 2}\in R^{C\times \frac{H}{2}\times \frac{W}{2}}\overset{\mathrm{reshape}}{⟶}AD_{2\times 2}^{\prime }\in R^{C\times \frac{HW}{4}},$$

$$\mathrm{and}\;AD_{4\times 4}\in R^{C\times \frac{H}{4}\times \frac{W}{4}}\overset{\mathrm{reshape}}{⟶}AD_{4\times 4}^{\prime }\in R^{C\times \frac{HW}{16}},$$

and concatenated them together as the face level representation *R*.

$$R=concat\left(AD_{1\times 1}^{\prime },\;AD_{2\times 2}^{\prime },\;AD_{4\times 4}^{\prime }\right)\;\;\in \;\;R^{C\times \frac{21\times H\times W}{16}}$$

(3) Learning the Difference Correlations

Considering difference correlations exist among different parts of the face (e.g., month region and nose region usually differ synchronously in the most expressive and most expressionless face images), we implanted a self-attention mechanism [44] to extract possible correlation representations $R_{q}$, $R_{k}$, and the original information remaining representation $R_{v}$ first.

(5) $$\begin{array}{c} R_{q}=ReLU\left(R\times W_{4}+b_{4}\right)\\ R_{k}=ReLU\left(R\times W_{5}+b_{5}\right)\\ R_{v}=ReLU\left(R\times W_{6}+b_{6}\right) \end{array}$$

where $W_{4}$, $W_{5}$, $W_{6}\in R^{\frac{21\times H\times W}{16}\times \frac{21\times H\times W}{16}}$ and $b_{4}$, $b_{5}$, $b_{6}\in R^{C\times \frac{21\times H\times W}{16}}$ are trainable parameters.

We applied the scaled dot product operation twice to obtain the matrix representation of the correlation between each pair of channels, and then got the weighted average representation *S*.

(6) $$S=Softmax\left(\frac{R_{q}\times R_{k}^{T}}{\sqrt{C}}\right)\times R_{v},$$

where *C* is the channels and $S\in R^{C\times \frac{21\times H\times W}{16}}$.

Finally, we reshaped *S* to one dimension:

$$S\in R^{C\times \frac{21\times H\times W}{16}}\overset{\mathrm{reshape}}{⟶}S^{\prime }\in R^{\frac{21\times C\times H\times W}{16}}$$

and used a fully connected layer to get the final face level representation.

(7) $$U_{fac}=ReLU\left(S^{\prime }\times W_{7}+b_{7}\right)$$

where $W_{7}\in R^{\frac{21\times H\times W}{16}\times m}$, $b_{7}\in R^{m}$ are trainable parameters, and *m* = 20 in the study.

#### 3.2.2. Action-Level Representation Learning

**Figure 3:**
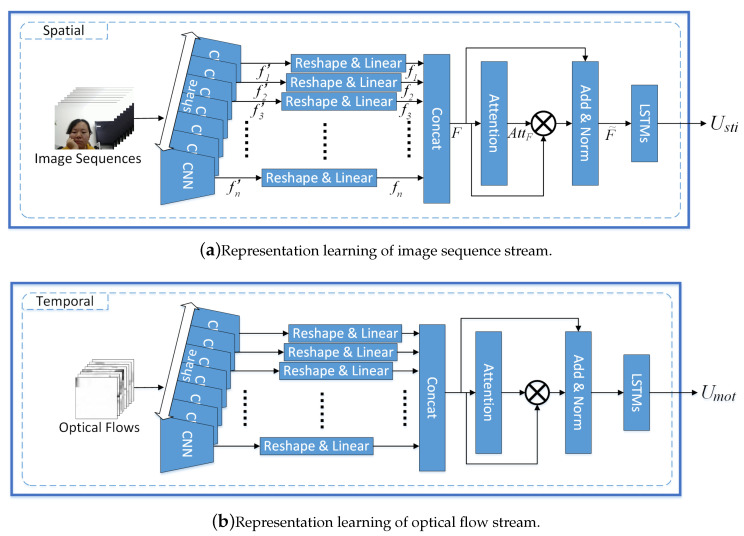
Action-level representation learning.

The learning of the action-level representation intends to grasp user’s action cues linked to stress. We explored the used of two streams derived from the user’s video *V*, which were (1) an image sequence $StiSeq\left(V\right)=\left(frame_{1},frame_{2},\cdots ,frame_{n}\right)$, denoting still image frames, and (2) an optical flow $MotSeq\left(V\right)=\left(mot1on_{1},motion_{2},\cdots ,motion_{n-1}\right)$, representing the motion between frames. We used the OpenCV warppers (https://github.com/feichtenhofer/gpu_flow) for optical flow extraction. Two networks were built for concurrently learning action-level representations. As both networks followed the same structure, we detail one of them in the following. Figure 3 shows the two steps of action-level representation learning based on the user’s still image sequence StiSeq(V).

Step 1: Learn and assign contribution weights to the still image frames.

Like the previous face images in Section 3.2.1, we applied Resnet [42] to the still images $frame_{1},frame_{2},\cdots ,frame_{n}\in StiSeq\left(V\right)$ to get respective basic feature maps $Resnet\left(frame_{1}\right),Resnet\left(frame_{2}\right),\cdots ,Resnet\left(frame_{n}\right)$$\in R^{2048\times 2\times 2}$.

To cut down the size of the feature maps, we executed the (2×2) average pooling to each basic feature map and lowered the 3-dimension to 1-dimension:

$$f_{i}^{\prime }=Pool\left(Resnet\left(frame_{i}\right)\right)\in \;\;R^{2048\times 1\times 1}\overset{\mathrm{reshape}}{⟶}f_{i}\in \;\;R^{2048}$$

where (1≤i≤n) and $\left(frame_{i}\in StiSeq\left(V\right)\right)$. We concatenated the obtained feature maps $f_{1},f_{2},\cdots ,f_{n}$ together:

$$F=[f_{1},f_{2},\cdots ,f_{n}]$$

We computed a contribution distribution matrix $Att_{F}$, which represents the importance and contribution of each still frame.

(8) $$\begin{array}{ccc} F^{\prime } & = & ReLU\left(F\times W_{1}+b_{1}\right)\;\;\;\in \;R^{n} \end{array}$$

(9) $$\begin{array}{ccc} Att_{F} & = & Softmax\left(W_{2}\times F^{\prime }+b_{2}\right)\;\;\;\in \;R^{n} \end{array}$$

where $W_{1}\in R^{2048\times 1}$, $W_{2}\in R^{n\times n}$, and $b_{1},b_{2}\in R^{n}$ are trainable parameters.

In this way, we could bind the still frames with respective contribution weights through applying element-wise multiplication with residual connection.

(10) $$\tilde{F}=Att_{F}\times F+F\;\;\in \;R^{n\times 2048}$$

Step 2: Learn the action-level representation based on the sequence of the weighted still image frames.

We presented $\tilde{F}\in R^{n\times 2048}$ in a frame-wise representation $\tilde{F}=\left({\tilde{F}}_{1},{\tilde{F}}_{2},\cdots ,{\tilde{F}}_{n}\right)$, where ${\tilde{F}}_{i}\in R^{1\times 2048}$.

We fed these weighted frames into LSTMs for sequential modeling, with an aim to capture the sequential action information.

(11) $$h_{t},\;c_{t}=LSTM\left({\tilde{F}}_{t},h_{t-1},c_{t-1}\right),$$

where $h_{t}$ and $c_{t}$ respectively represent the hidden state and the cell state at the *t*-th time in the sequence $\left({\tilde{F}}_{1},{\tilde{F}}_{2},\cdots ,{\tilde{F}}_{n}\right)$. With the last state $c_{n}$ out of the LSTMs, we generated the action-level representation based on the still image frame sequence:

(12) $$\begin{array}{c} U_{sti}=ReLU\left(W_{3}\times c_{n}+b_{3}\right), \end{array}$$

where $U_{sti}\in R^{m}$ is the output, $W_{3}\in R^{m\times 2048}$ and $b_{3}\in R^{m}$ are trainable parameters.

In a similar manner, we could get $U_{mot}$ as the action-level representation based on the motion sequence in the video (as shown in Figure 3b).

#### 3.2.3. Integrating Face- and Action-Level Representations for Stress Detection

We designed a weighted integration with local and global attention method to learn the contributions of face-level and action-level streams and incorporated them as weights for stress identification.

**Figure 4:**
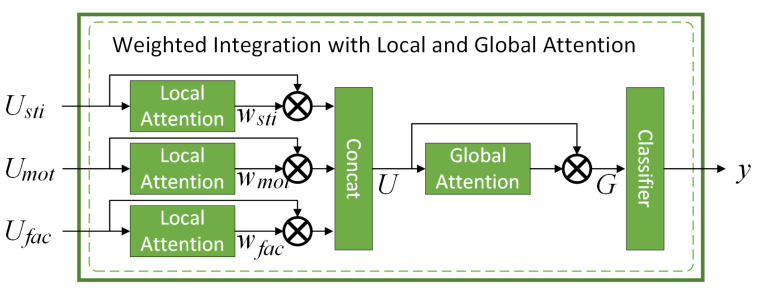
Integrating face- and action-level representations for stress detection.

As shown in Figure 4, the three inputs $U_{sti}$, $U_{mot}$, and $U_{fac}$ went through the respective local attention layer with three weights $U_{sti}$, $U_{mot}$, and $U_{fac}$ being derived.

(13) $$\begin{array}{ccc} w_{sti} & = & ReLU(W_{8}\times U_{sti}+b_{8}) \end{array}$$

(14) $$\begin{array}{ccc} w_{mot} & = & ReLU(W_{9}\times U_{mot}+b_{9}) \end{array}$$

(15) $$\begin{array}{ccc} w_{fac} & = & ReLU(W_{10}\times U_{fac}+b_{10}) \end{array}$$

where $W_{8}$, $W_{9}$, $W_{10}\in R^{1\times m}$, and $b_{8}$, $b_{9}$, $b_{10}\in R^{1}$ are trainable parameters.

We concatenated the three weighted streams into one, which was then passed through a global attention layer, and arrived at the final classification layer for stress identification.

(16) $$\begin{array}{ccc} U & = & \left[w_{sti}\times U_{sti},w_{mot}\times U_{mot},w_{fac}\times U_{fac}\right]\;\;\in R^{3m} \end{array}$$

(17) $$\begin{array}{ccc} G & = & ReLU\left(W_{11}\times U+b_{11}\right)\times U+U\;\;\in R^{3m} \end{array}$$

(18) $$\begin{array}{ccc} y & = & Softmax(W_{12}\times G) \end{array}$$

where $W_{11}\in R^{3m}$, $b_{11}\in R^{3m}$, and $W_{12}\in R^{classnum\times 3m}$ are trainable parameters, and classnum = 2 in this paper.

## 4. Results

### 4.1. Evaluation Metrics

We evaluated our proposed TSDNet on the collected video dataset. We compared the performance of TSDNet and several existing methods in terms of four widely used metrics: F1-Score, precision, recall, and accuracy, where

F1-Score is an often-used metric in the fields of information retrieval and natural language processing. It is interpreted as the weighted average of precision and recall. It is a measure of the statistical accuracy of the model given as follows:

$$F1-Score\left(precision,recall\right)=\frac{2\times Recall\times Precision}{Recall+Precision}$$

Recall is the measure of the ability of the model to select instances of a certain class from the dataset. It is the sensitivity of the model defined as:

$$Recall=\frac{TP}{TP+FN}$$

where TP is the number of true-positive classifications and FN is the number of false-negative classifications.

Precision is the measure of the accuracy if a specific class is classified:

$$Precision=\frac{TP}{TP+FP}$$

where FP is the number of false-positive classifications.

Accuracy is the measure of the accuracy over all the classes:

$$Accuracy=\frac{TP+TN}{TP+FP+TN+FN}$$

### 4.2. Implementation Details

We followed the uniform random distribution U (−0.001,0.001) to initialize all the trainable parameters in the model. The learning rates were initialized as 0.01. All the learning rates were divided by 2 every 15 epochs. The batch size was 64. We used 120 epochs to train our stress detection model. The optimization process fine-tuned all the layers with stochastic gradient descent (SGD) through a weight decay of 0.01 with a momentum of 0.9.

As the study focused on low-end video camera without thermal spectrums, we compared the performance of our method with the following two categories of video-based stress detection approaches.

(1) Action Units (AUs) based: (1) The Dependent Model [20] extracted 17 different Action Units (AUs) from videos of people’s facial expressions, and applied different classifiers (including Random Forest, Gaussian Naive Bayes, and Decision Tree) to detect stress. (2) FDASSNN [21] also employed the Facial Action Coding System (FACS) to extract facial action units as features, and then constructed a three-layered neural network architecture to detect Depression Anxiety Stress Scale levels.

(2) Facial Cues (FCs) based: [13] was a representative approach, which extracted a set of facial signs including mouth activity, head motion, heart rate, blink rate, and eye movements from different facial regions to classify one’s stress and anxiety level.

Our implementation was based on the deep learning framework PyTorch. All the experiments were conducted on two NVIDIA GTX Titan X GPU with 24 GB on-board memory in total.

### 4.3. Performance Evaluation

Three sets of experiments were conducted to evaluate the performance of TSDNet in stress detection, as well as its design details, including face-level, action-level, and integration local and global attention mechanisms and different integration strategies.

#### 4.3.1. Experiment 1: Performance Comparison

Table 2 shows the performance of our TSDNet method compared with two other categories of video-based stress detection methods. TSDNet outperformed the best among all the methods with the highest accuracy 85.42% and F1-Score 85.28%. In comparison, the Action Units based approach (FDASSNN) achieved up to 74.11% of detection accuracy and 73.71% of F1-Score, and the Facial Cues based approach (FC) had the lowest accuracy 46.64% and F1-Score 42.61%. The results demonstrated the feasibility and advantages of using deep learning to analyze one’s face and action motions over the traditional hand-crafted feature engineering strategy.

From the TSDNet’s confusion detection matrix shown in Table 3, we can find that TSDNet worked evenly well in stress detection.

Moreover, considering the motions of both face and action in TSDNet could effectively improve the detection accuracy and F1-Score of that considering only face or action method by over 7%.

#### 4.3.2. Experiment 2: Effectiveness of Attention Mechanisms in TSDNet

**Figure 5:**
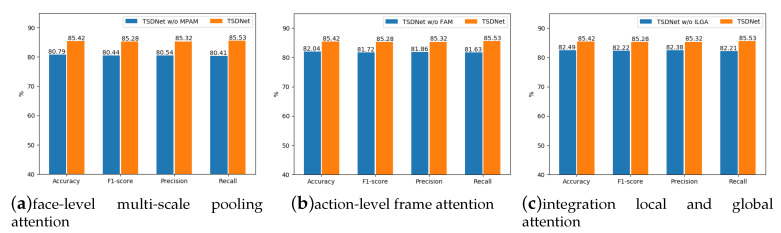
Effectiveness of attention mechanisms in TSDNet.

The second experiment investigated the effectiveness of different attention mechanisms (including face-level multi-scaled pooling attention, action-level frame attention, and integration local and global attention), which we designed and incorporated in TSDNet. We conducted three ablation studies which respectively removed the attention mechanisms from TSDNet. From the results presented in Figure 5, we can find that without the face-level multi-scale pooling attention, action-level frame attention, and integration local and global attention, the detection accuracy and F1-score respectively drop about 5%, 3%, and 3%, respectively. The results verify the effectiveness of our designed attention mechanisms for stress detection.

In the face-level multi-scaled pooling attention, we took an average pooling with kernel size of (1 × 1), (2 × 2), and (4 × 4). We compared the different pooling combination methods, i.e., (1 × 1) + (2 × 2) pooling, (1 × 1) + (2 × 2) + (4 × 4) pooling, and (1 × 1) + (2 × 2) + (4 × 4) + (8 × 8) pooling. As shown in Table 4, the pooling (1 × 1) + (2 × 2) + (4 × 4) achieved the best result, and more or less pooling might lead to a similar decline in accuracy and F1-Score.

#### 4.3.3. Experiment 3: Effectiveness of the Stream Weighted Integration Method in TSDNet

**Figure 6:**
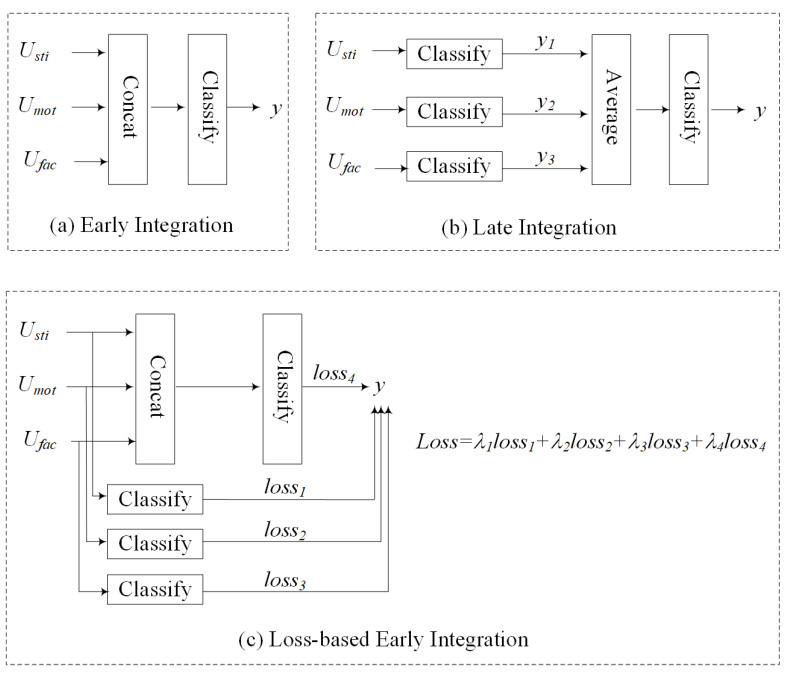
Three other integration methods: Early integration, loss-based early integration, and later integration. In the loss-based early integration $Loss=\lambda_{1}loss_{1}+\lambda_{2}loss_{2}+\lambda_{3}loss_{3}+\lambda_{4}loss_{4}$, $\lambda_{1}$, $\lambda_{2}$, $\lambda_{3}$, and $\lambda_{4}$ are set as 0.2, 0,2, 0,2, and 0,4, respectively.

We compared our designed stream weighted integration with local and global attention method with three other integration approaches, which are early integration, loss-based early integration, and later integration, as illustrated in Figure 6.

Table 5 shows the performance of different integration methods in stress detection. The designed stream weighted integration method used in TSDNet achieved the best result with 85.42% in accuracy and 85.28% in F1-Score. It verified that in different scenes $U_{sti}$, $U_{mot}$ and $U_{fac}$ contributed differently, and the stream weighted integration with local and global attention method could automatically distribute the weights of the three streams under different situations.

## 5. Conclusions

In this paper, we presented a video-based Two-leveled Stress Detection Network (TSDNet), which integrates face-level detector and action-level detector to understand facial expressions and action motions for stress identification. In particularly, we designed a face-level multi-scale pooling attention mechanism and an action-level frame attention mechanism. The former employed the multi-scaled average pooling with different kernel sizes to grasp stress-related facial features, and the latter focused on key body movement frames related to stressed states. A stream weighted integrator with local and global attention was used to fuse the results from face- and action-level detectors. We built a video dataset containing 2092 labeled video clips, and evaluated the performance of TSDNet on the data set. The experimental results show that TSDNet outperformed the existing hand-crafted feature-engineering strategies, and integrating face-level and action-level detectors could improve detection accuracy and F1-Score by over 7%.

In future work, we plan to add the audio stream into the framework to explore the audio–video methods for stress detection.

## Figures and Tables

**Table 1:** Video samples used for training, validation, and testing.

Divisions	Video Samples	#Training	#Validation	#Testing	#Total
1	Stressed	595	173	152	920
	Unstressed	746	214	212	1172
	Total	1341	387	364	2092
2	Stressed	590	171	159	920
	Unstressed	741	228	203	1172
	Total	1331	399	362	2092
3	Stressed	560	182	178	920
	Unstressed	739	212	221	1172
	Total	1299	394	399	2092

**Table 2:** Performance comparison among two-leveled stress detection network (TSDNet) and two other categories of video-based stress detection methods.

Category	Method	Accuracy	F1-Score	Precision	Recall
FCs-based	FC	46.64%	42.61%	52.47%	49.98%
AUs-based	Dependent Model (Random Forest)	67.17%	66.82%	66.97%	67.14%
	Dependent Model (Gaussian Naive Bayes)	70.46%	70.28%	71.39%	71.08%
	Dependent Model (Decision Tree)	68.77%	68.35%	68.36%	68.41%
	FDASSNN	74.11%	73.71%	74.00%	74.06%
TSDNet	Face only	78.62%	78.17%	78.31%	77.97%
	Action only	78.40%	78.13%	78.20%	78.60%
	Face + Action	**85.42%**	**85.28%**	**85.32%**	**85.53%**

**Table 3:** Confusion matrix of TSDNet in stress detection.

	Detected	Stressed	Unstressed
Actual			
Stressed		86.91%	13.09%
Unstressed		15.72%	84.28%

**Table 4:** Performance of pooling sizes in the face-level multi-scaled pooling attention.

No	Pooling Combination Methods				Accuracy	F1-Score	Precision	Recall
	(1 × 1)	(2 × 2)	(4 × 4)	(8 × 8)				
1	✓	×	×	×	81.42%	81.13%	81.30%	81.09%
2	✓	✓	×	×	82.13%	81.82%	81.88%	81.87%
3	✓	✓	✓	×	85.42%	85.28%	85.32%	85.53%
4	✓	✓	✓	✓	83.37%	83.03%	83.34%	83.02%

**Table 5:** Performance of different integration methods.

Integration Method	Accuracy	F1-Score	Precision	Recall
Early Integration	82.49%	82.22%	81.86%	81.63%
Loss-based Early Integration	84.44%	84.24%	84.41%	84.31%
Late Integration	82.20%	82.04%	82.05%	82.31%
Weighted Integration with Local and Global Attention	85.42%	85.28%	85.32%	85.53%
